# Acetylcholinesterase Inhibitors Assay Using Colorimetric pH Sensitive Strips and Image Analysis by a Smartphone

**DOI:** 10.1155/2017/3712384

**Published:** 2017-02-13

**Authors:** Adam Kostelnik, Alexander Cegan, Miroslav Pohanka

**Affiliations:** ^1^Faculty of Chemical Technology, University of Pardubice, Studentska 95, 53210 Pardubice, Czech Republic; ^2^Faculty of Military Health Sciences, University of Defense, Trebesska 1575, 50001 Hradec Kralove, Czech Republic; ^3^Department of Geology and Pedology, Mendel University in Brno, Brno, Czech Republic

## Abstract

Smartphones are widely spread and their usage does not require any trained personnel. Recently, smartphones were successfully used in analytical chemistry as a simple detection tool in some applications. This paper focuses on immobilization of acetylcholinesterase (AChE) onto commercially available pH strips with stabilization in the gelatin membrane. AChE degrades acetylcholine into choline and acetic acid which causes color change of acid-base indicator. Smartphone served as a tool for measurement of indicator color change from red to orange while inhibitors blocked this process. AChE inhibitors were measured with limits of detection, 149 nM and 22.3 nM for galanthamine and donepezil, respectively. Organic solvents were measured for method interferences. Measurement procedure was performed on 3D printed holder and digital photography was evaluated using red-green-blue (RGB) channels. The invented assay was validated to the standard Ellman's test and verified on murine plasma samples spiked with inhibitors. We consider that the assay is fully suitable for practical performance.

## 1. Introduction

AChE is an enzyme splitting the neurotransmitter acetylcholine in cholinergic synapsis into choline and acetic acid [1]. Sensitivity of AChE to neurotoxic compounds (anti-Alzheimer's drugs, pesticides, and nerve agents) can be use in their measurement [2]. Commonly used method is based on reaction of thiocholine, formed from acetylthiocholine during enzymatic hydrolysis, with Ellman's reagent producing yellow 5-thio-2-nitrobenzoate measurable by spectrophotometry in 412 nm [3]. Despite broad use, this method has some drawbacks like instability of Ellman's reagent and hemoglobin interference [2, 4, 5]. Furthermore, there is possibility of measuring enzyme activity electrochemically [6, 7]. Colorimetric detection can be also based on pH measurement. Many acid-base indicators are known for this purpose, when phenol red was used in our work previously [8, 9]. Different techniques for enzyme immobilization and matrix like gelatin were described as well [10–13]. Simple physical immobilization of enzyme onto cellulose and stabilization into gelatin matrix was successfully used for preparation of biosensor in an application where AChE was embedded into gelatin membrane on a paper matrix and performed for the assay of neurotoxic compounds [14, 15]. In the cited papers, there was no, however, made evaluation of enzyme activity by a camera because a naked eye assay and another type of substrate were preferred. Gelatin provides good properties for enzyme immobilization combined with biocompatibility and zero toxicity [16]. Modern mobile phones dispose high resolution cameras which gives them ability to serve as a tool for diagnostics [17, 18]. Previously mobile phones have been used for some applications in analytical chemistry [19, 20]. This paper deals with preparation of biosensor based on commercial pH strips with immobilized AChE and stabilized in gelatin membrane. Performance of the biosensor was verified on neurotoxic compounds. This approach offers easy way for AChE inhibitors determination, especially if we considered well-established spectrophotometric assay; for the presented method here, no special equipment or trained personnel are required. There is also innovation in evaluation of color based reaction compared to previously described methods. The major advantage of our assay is based on the opportunity to link it to a smartphone which is considered as the detector device providing wide availability to less equipped laboratories and for field tests without any expensive gear.

## 2. Material and Methods

### 2.1. Materials and Devices

Acetylcholinesterase from electric eel (≥1000 units/mg protein), acetylcholine chloride (AChCl), acetylthiocholine chloride (ATChCl), 5,5′-dithiobis(2-nitrobenzoic acid) (DTNB), donepezil hydrochloride monohydrate, galanthamine hydrobromide, tetraisopropyl pyrophosphoramide (iso-OMPA), phosphate buffer saline (PBS) pH 7.4, dimethyl sulfoxide (DMSO), and isopropyl alcohol were purchased from Sigma-Aldrich (St. Louis, MO, USA); denatured ethanol and gelatin were supplied by PENTA (Prague, Czech Republic). Indicator strips pH-Fix 6.0–7.7 were obtained from Macherey-Nagel (Düren, Germany). Color change was detected by Sony Xperia MT27i with 5 Mpx camera and LED light using operation system Android 2.3.7., device version number 6.0.B.3.184 (Tokyo, Japan). For 3D print, 3D printer Prusa i3 from Prusa Research (Prague, Czech Republic) was used. Murine plasma samples were obtained from 20 female BALB/c mice which were purchased from Velaz (Unetice, Czech Republic). The mice were kept under standard ambient temperature and humidity 50 ± 10%. Light and dark periods lasted equally for 12 hours each. The mice were sacrificed in the age of 8 weeks by cutting of carotid under carbon dioxide narcosis and the blood was taken into tubes pretreated with lithium heparin (Dialab, Prague, Czech Republic) and centrifuged at 1,000 ×g for 5 minutes. Fresh plasma was kept at −80°C until use in the assay. The whole experiment was both permitted and supervised by ethical committee Faculty of Military Health Sciences (Hradec Kralove, Czech Republic).

### 2.2. Solutions Preparation

AChCl solutions were prepared in concentration range from 0.31 to 10 mM and placed in microtubes. Galanthamine solutions were prepared in concentration range from 1.6 to 25.00 *µ*M. Donepezil solutions were prepared in concentration range from 0.31 to 5.00 *µ*M. Each solution was prepared in PBS 7.4 and final concentration in microtube was 10 times lower. Gelatin was prepared in 1% concentration by stirring of 10 mg of gelatin in 1 ml of water for 20 min. All solutions for Ellman's assay were prepared in PBS 7.4. DTNB solution was prepared in concentration 1 mM and ATChCl in 10 mM. Concentration range of galanthamine was from 62.5 to 100 *µ*M and 13 to 20 *µ*M in case of donepezil. Final concentrations in cuvette were 10-fold less concentration of ATChCl and 40-fold less in case of galanthamine or donepezil. Iso-OMPA was prepared in PBS 7.4 in concentration 1 mM; final concentration in plasma samples was 0.1 mM.

### 2.3. Preparation of pH Strips with AChE

To pH strips 10 *µ*l of AChE (activity for acetylthiocholine 1.73 × 10^−8^ mol/s/*µ*l) was added and let to dry in laboratory temperature. Then pH strips were covered by 10 *µ*l of 1% gelatin. After drying in laboratory temperature pH strips were stored in 4°C until used in the assay.

### 2.4. Preparation of 3D Printed Holder

**Figure 1:**
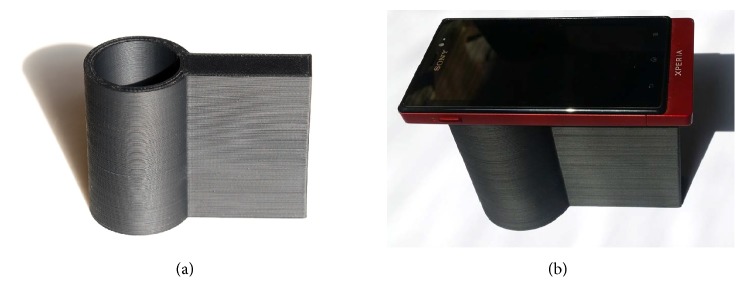
Tube shaped holder printed by 3D print technology (a) and the holder with a smartphone adjusted on the hole to provide photographs by an integrated camera.

Holder was created in Autodesk 123D Design (open source software). 3D printer setup was as follows: acrylonitrile butadiene styrene shaped in 3 mm filaments was used as material, nozzle temperature was at 285°C and bottom temperature at 100°C, and individual deposited layers were 0.1 mm thick. Size of holder was 80 mm in height and 105 mm in length and inner diameter of tube was set to 40 mm (Figure 1).

### 2.5. Smartphone Assay

The smartphone assay was made in the following way: 450 *µ*l of PBS pH 7.4 and 50 *µ*l of 10 mM AChCl solution were added to microtubes and strip was put into it. After incubation of 15 min, excess of reaction medium was drained and picture of the still wet pH strip was taken. During photographing, the camera was placed on the 3D printed holder and the strip inside; hence, no outer light influenced the photography and integrated LED light was the only one source. Distance between the strip and camera was also constant just due to the holder.

### 2.6. Ellman's Assay

To standard cuvette 400 *µ*l of DNTB solution, 25 *µ*l of AChE, 475 *µ*l of PBS, and 100 *µ*l of ATChCl were added. Absorbance was measured in 412 nm immediately and after incubation of 2 min.

### 2.7. Data Processing

RGB color values were obtained by processing of photography in GIMP 2.8.16 (open source software) using Color Picker function. ΔColor intensity was obtained as follows: intensity of strip before reaction – intensity of strip after the reaction. This difference corresponds to AChE activity in different concentration of used substrate or inhibitor. *K*_*M*_ value for AChE and AChCl substrate was calculated using nonlinear curve fitting by Hill function with coefficient of cooperativity *n* = 1. Limit of detection was calculated as signal to noise ratio equal to three. For these purposes, Origin software 8 PRO (OriginLab, Northampton, MA, USA) was used.

## 3. Results and Discussion

**Figure 2:**
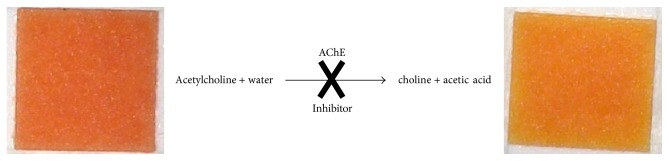
pH sensitive method using AChE principle.

AChE splitting acetylcholine into choline and acetic acid resulted in decreasing pH of medium. Our method is based on color change of indicator in pH sensitive zone of pH strip from red to orange (Figure 2). During photography processing color change was observed in green channel while red and blue ones were without change.

### 3.1. Gelatin Optimization

**Figure 3:**
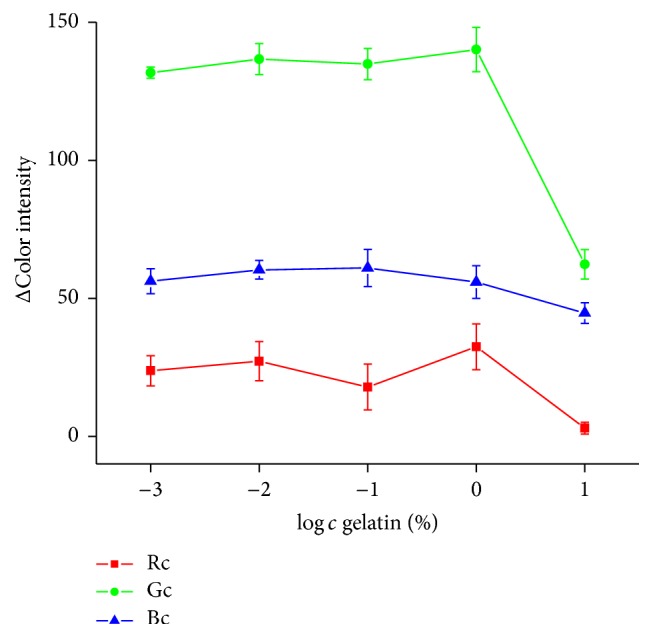
Gelatin optimization. Drop in ΔColor intensity indicates that pH strip did not change into red color. Error bars represent standard error of the mean for *n* = 3. Rc = red channel, Gc = green channel, and Bc = blue channel.

Amount of gelatin was tested in 0%, 0.001%, 0.01%, 0.1%, 1%, and 10% concentration. The strips were covered with 10 *µ*l of gelatin in tested concentration and dipped into PBS 7.4 and color change of strip into red was observed after 5 min. Gelatin in 10% concentration did not allow us to enter buffer to pH sensitive zone and color did not change into red one while it stayed orange which was represented by decreasing of color intensity (Figure 3). Color change into red was held while using gelatin in 1% and less concentration, but for further measurement 1% gelatin was chosen just for the better stabilization effect for AChE.

### 3.2. Time Optimization

**Figure 4:**
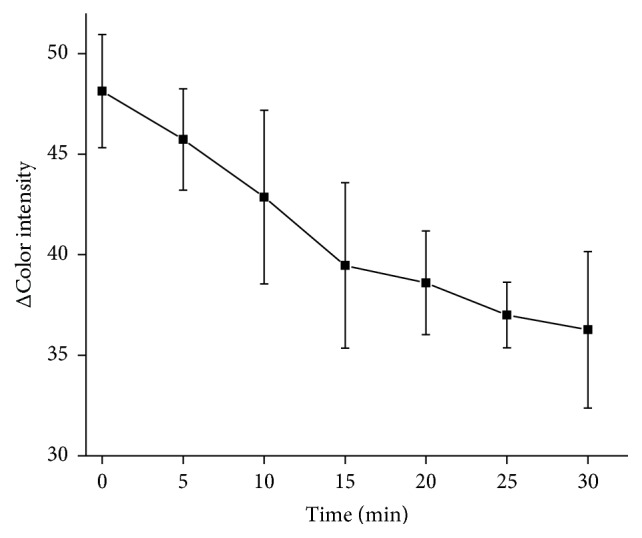
Optimization of pH strip incubation time with ATChCl as a substrate. Error bars represent standard error of the mean for *n* = 3.

Strip with AChE and 1% gelatin was incubated with 1 mM AChCl. Incubation time with AChCl was observed in 5 min intervals from 0 to 30 min. The biggest color change was held up to 15 min. Over this time color change was not significant for longer incubation time (Figure 4).

### 3.3. Substrate Measurement

**Figure 5:**
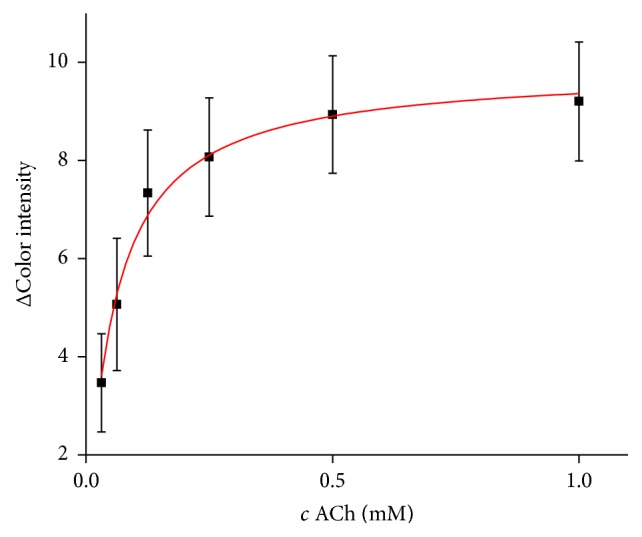
Saturation curve for AChE and AChCl as a substrate. Error bars represent standard error of the mean for *n* = 3.

Saturation curve for AChE and AChCl as a substrate was measured in concentration range from 0.031 to 1.0 mM and *K*_*M*_ value was calculated as described above to 54.26 *µ*M (Figure 5) while 73.9 *µ*M was reported by Xu and coworkers [24]. *K*_*M*_ value however depends on type of used buffer as proved by Wille et al. using human AChE isolated from erythrocytes and achieved *K*_*M*_ equal to 71.4 *µ*M in MOPS buffer, 98.2 *µ*M in PBS buffer, 0.1 mM in Tyrode buffer, and 0.122 mM in Tris buffer when pH was set to 7.4 [25]. There are also big differences between organisms as shown by Shaonan et al. who worked with AChE isolated from fish species and found out values above 0.1 mM [26] and by Jiang et al. who reported *K*_*M*_ to be 63.85 *µ*M when measured with mosquito AChE [27]. Recombinant enzymes exhibit slightly higher *K*_*M*_ values compared to wild types as showed in experiments with mice AChE carried out by Boyd et al. who found out *K*_*M*_ to be 46 *µ*M in wild type and 58 *µ*M for recombinant AChE [28].

### 3.4. Inhibitors Measurement

**Figure 6:**
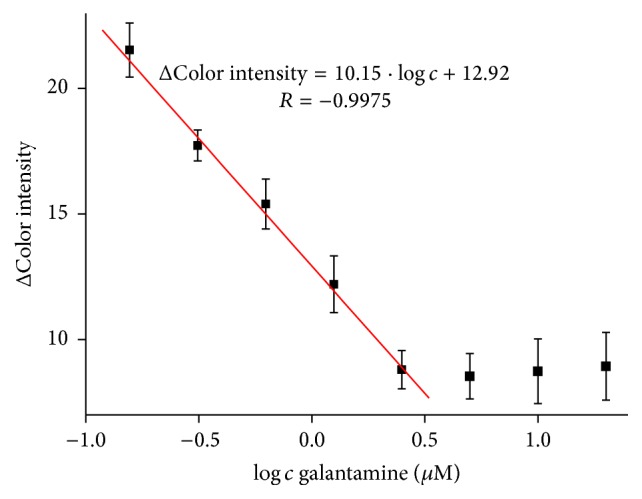
Calibration curve of galanthamine; concentration is given in logarithm. Error bars represent standard error of the mean for *n* = 3.

AChE is sensitive to neurotoxic compounds like drugs, nerve agents, or pesticides. Some of these compounds are widely used in treatment of Alzheimer disease [29]. Galantamine and donepezil can be examples for the currently available drugs [30]. We performed calibration curve of galanthamine in concentration range from 0.156 to 2.5 *µ*M with limit of detection calculated to 149 nM and quantification limit of 0.5 *µ*M was achieved (Figure 6). Linearity of the assay is limited to 2.5 *µ*M when higher concentrations appeared to be indistinguishable. Comparing to the standard Ellman's assay, it is only 10 times higher detection limit, when 18.3 nM of galanthamine was achieved in our experiment. From previously published methods for galanthamine measurement based on AChE inhibition we can conclude that our method is competitive [21, 22]. Although detection limits are similar, there is advantage in fabrication time of pH strip with immobilized AChE which is not time consuming.

**Figure 7:**
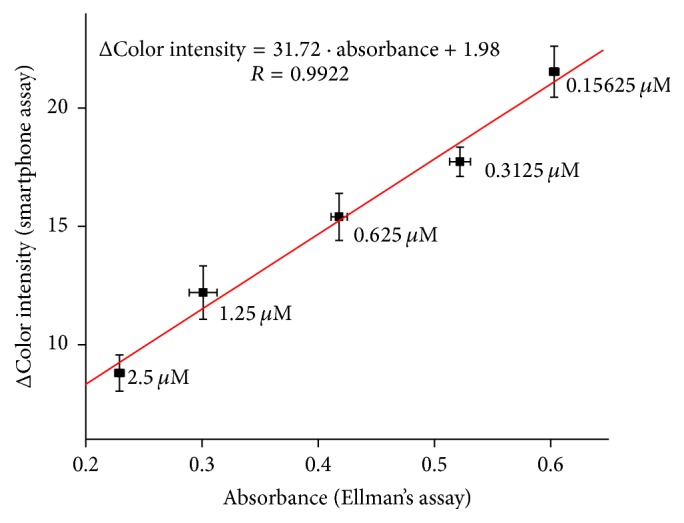
Validation of galanthamine assay compared to standard Ellman's assay. Error bars for smartphone assay represent standard error of the mean and for Ellman's assay standard deviation for *n* = 3.

Validation of method for galanthamine measurement was done using standard Ellman's assay with correlation coefficient of 0.9922 (Figure 7).

**Figure 8:**
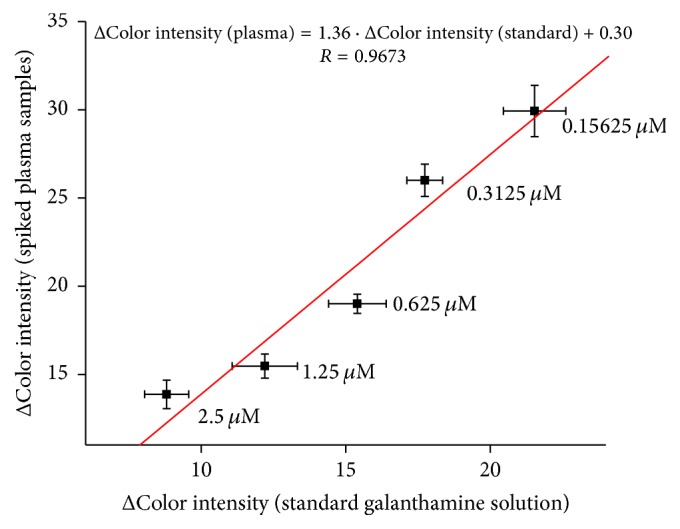
Verifying of galanthamine assay in plasma samples compared to standard galanthamine solution. Error bars represent standard error of the mean for *n* = 3.

Method was verified using murine plasma. Plasma samples were pretreated by iso-OMPA, selective inhibitor of butyrylcholinesterase [31], which naturally occurs in plasma and has ability to split acetylcholine. Then galanthamine was spiked into plasma samples in appropriate concentrations and smartphone assay has been performed (Figure 8). For measurement with plasma PBS buffer had to be replaced by water, because plasma strongly buffered itself; then PBS buffer was not required.

**Figure 9:**
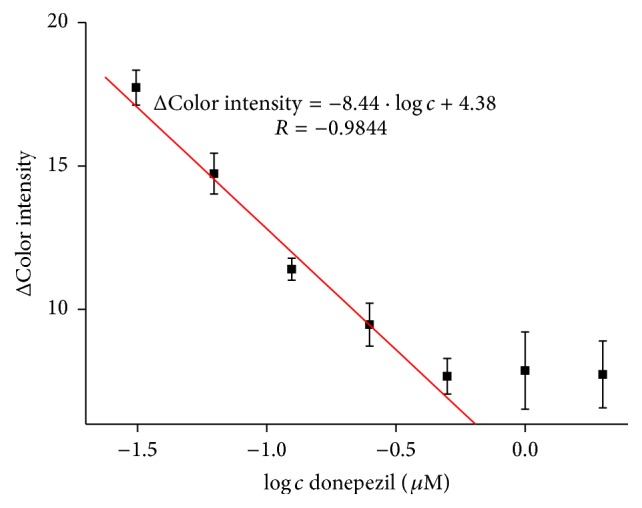
Calibration curve of donepezil; concentration is given in logarithm. Error bars represent standard error of the mean for *n* = 3.

In literature, there are plenty of references about determination of donepezil by chromatography techniques [32, 33]. Likewise, there are electrochemical methods for donepezil measurement with similar detection limits as our method [23, 34] and some spectrophotometric methods for quantification of donepezil in pharmaceuticals or human plasma [35, 36]. However, no evidence about determination of donepezil via inhibition of AChE was found. Donepezil calibration curve was done in concentration range from 0.031 to 0.50 *µ*M and limit of detection equal to 22.3 nM was achieved while limit of quantification was found to be 0.2 *µ*M (Figure 9). Also, above calibration range there is limited linearity. For comparison, detection limit achieved by Ellman's assay in this experiment was equal to 3.82 nM.

**Figure 10:**
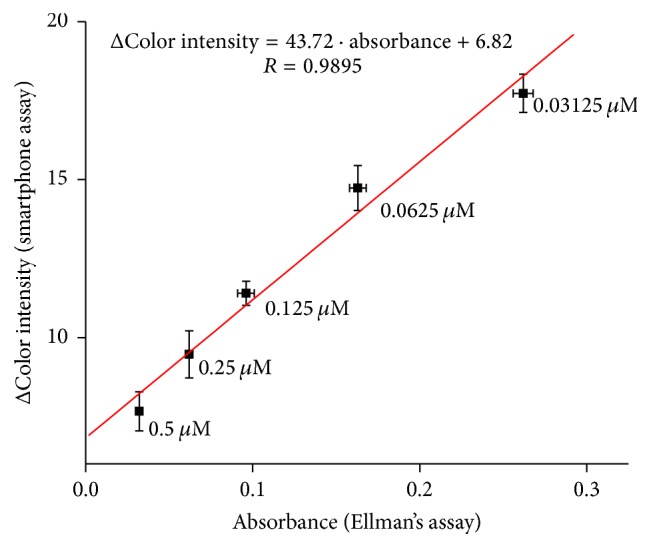
Validation of donepezil assay compared to standard Ellman's assay. Error bars for smartphone assay represent standard error of the mean and for Ellman's assay standard deviation for *n* = 3.

Validation of method for donepezil measurement was performed using standard Ellman's assay with correlation coefficient equal to 0.9895 (Figure 10).

**Figure 11:**
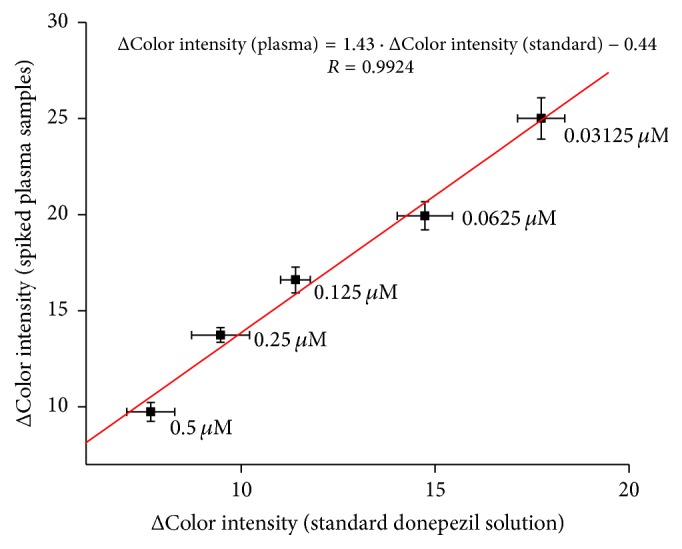
Verifying of donepezil assay in plasma samples compared to standard donepezil solution. Error bars represent standard error of the mean for *n* = 3.

Verifying of donepezil in plasma samples was done as well. Donepezil was spiked in appropriate concentrations into pretreated plasma samples and smartphone assay was performed (Figure 11). PBS buffer was also replaced by water for reason mentioned in the principle based on pH change where a strong buffering could interfere. It appears that both measurements are in a good correlation.

### 3.5. Organic Solvents

**Figure 12:**
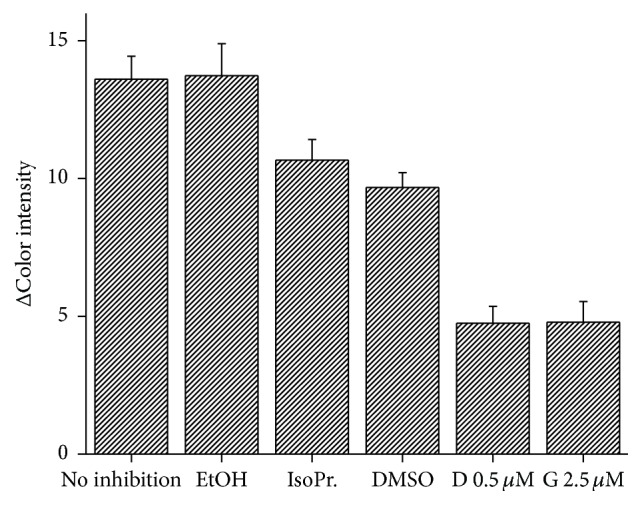
Method interferences by organic solvents. EtOH = ethanol, IsoPr. = isopropyl alcohol, DMSO = dimethyl sulfoxide, D 0.5 *µ*M = donepezil in 0.5 *µ*M concentration, and G 2.5 *µ*M = galanthamine in 2.5 *µ*M concentration. Error bars represent standard error of the mean for *n* = 3.

Because AChE activity can be reduced by organic solvents in small concentration [37], we decided to consider them as possible interferents in the AChE based assay. For this purpose ethanol, isopropyl alcohol, and dimethyl sulfoxide were tested, all in 5% concentration. Influence of DMSO to AChE activity has been investigated before and results showed that DMSO slightly decreases enzyme activity which was confirmed in our work [38]. Ethanol has been reported to decrease AChE activity [37, 39]; however, there is evidence that at low concentration it can enhance enzyme activity [38, 40]. No activity change against uninhibited enzyme was observed in our work. Isopropyl alcohol as well as ethanol can drop AChE activity [41] which is in agreement with our results. To compare used inhibitors, donepezil and galanthamine are included in graph (Figure 12).

## 4. Conclusion

Here, the presented method proved its ability for determination of neurotoxic compounds with promising limits of detection for galanthamine and donepezil, 149 nM and 22.3 nM, respectively. Assay was successfully validated to the standard Ellman's spectrophotometric test and showed feasibility of measurement in plasma samples. Additionally, no specialized equipment and trained personnel are required; combined with low cost, portability, easy preparation, and miniaturization are considered as big advantages of the here invented method. Comparison of presented method with standard Ellman's assay and literature is given in Table 1.

## Figures and Tables

**Table 1:** Comparison of presented assay with Ellman's assay and literature.

	Detection limit	Fabrication time	Assay time	Necessary equipment
Presented smartphone assay	Galanthamine: 149.1 nM Donepezil: 22.3 nM	Aprox. 1 hour	15 min	Smartphone
Standard Ellman's assay	Galanthamine: 18.3 nM Donepezil: 3.82 nM	NA	10 min	Spectrophotometer
Spectrophotometric assay [21]	Galanthamine: 0.05 nM	Aprox. 3.5 hours	Aprox. 20 mn	Spectrophotometer
Potentiometric assay [22]	Galanthamine: 5.4 nM	Aprox. 10 hours	Aprox. 10 min	Electrodes, potentiometer
Square-wave voltammetry [23]	Donepezil: 151 nM	NA	Aprox. 20 min	Electrodes, electrochemical analyser
